# Sex Differences in the Relationship of Serum Vitamin B1 and B12 to Dementia Among Memory Clinic Outpatients in Japan

**DOI:** 10.3389/fnagi.2021.667215

**Published:** 2021-04-09

**Authors:** Ayako Miki, Ryuta Kinno, Hirotaka Ochiai, Satomi Kubota, Yukiko Mori, Akinori Futamura, Azusa Sugimoto, Takeshi Kuroda, Hideyo Kasai, Satoshi Yano, Sotaro Hieda, Akatsuki Kokaze, Kenjiro Ono

**Affiliations:** ^1^Department of Hygiene, Public Health and Preventive Medicine, Showa University School of Medicine, Tokyo, Japan; ^2^Department of Neurology, School of Medicine, Showa University, Tokyo, Japan

**Keywords:** dementia, cognitive impairment, sex differences, vitamin B1 (thiamine), vitamin B12 (cobalamin), memory clinic outpatients

## Abstract

Dementia and cognitive impairment are considered to be one of the biggest social and medical problems. While there is a definite relationship between vitamin B and cognitive decline, this has yet to be fully assessed with regard to sex differences. Thus, the present study investigated the relationship of vitamin B1 or vitamin B12 with dementia in accordance with the sex in 188 patients who visited the Memory Clinic at Showa University Hospital in Japan from March 2016 to March 2019. Cognitive function was tested by the Japanese version of the Mini-Mental State Examination (MMSE) and Hasegawa Dementia Scale-Revised (HDS-R). Blood tests were performed to measure the vitamin levels. Logistic regression analysis was used to calculate the odds ratio (OR) for dementia and the 95% confidence interval (CI). Compared to the highest vitamin group (third tertile), the lowest vitamin group (first tertile) exhibited a significantly increased OR for dementia defined by MMSE for vitamin B1 (OR:3.73, 95% CI:1.52–9.16) and vitamin B12 (2.97, 1.22–7.28) among women. In contrast, vitamin levels were not significantly associated with dementia determined by MMSE in men. These findings were similar even when dementia was defined by HDS-R. The present study suggests that vitamin B1 plays a role in preventing development of dementia in women. Future longitudinal studies will need to be undertaken in order to examine whether decreasing vitamin levels occur before or after cognitive impairment, and whether maintaining a higher vitamin level can prevent a worsening of cognitive function and the development of dementia.

## Introduction

Recently, dementia and cognitive impairment, which results from multiple factors including Alzheimer’s disease, has become an increasingly large social issue. With the aging of society, the number of dementia patients is increasing, which has led to heavy economic and healthcare burdens (Rice et al., 1993; Cantarero-Prieto et al., 2019). In 2015, there were approximately 46.8 million people with dementia worldwide, with the global cost estimated to be 604 billion dollars in 2010 (Alzheimer’s Disease International, 2015). Similarly in Japan, over 4.6 million patients had dementia in 2012, while 4 million people were designated as having Mild Cognitive Impairment (MCI) (Cabinet Office Japan, 2017). Although there have been a large number of research studies worldwide that have examined the prevention and treatment of dementia, definitive treatments have yet to be discovered and implemented (Saji et al., 2016).

Many previous studies have investigated the relationship between vitamins and dementia (O’Leary et al., 2012; Gibson et al., 2016; Hughes et al., 2017; Travica et al., 2017; Balbaloglu and Tanik, 2019). However, the relationship between the vitamin B level and cognitive decline has yet to be fully assessed, especially with regard to differences associated with the sex. Moreover, there have been few studies that have examined the association between vitamin B and dementia in Japan. Vitamin B1 (thiamine) is one of the most well-known nutritional deficiencies that can cause various diseases such as beriberi and Wernicke-Korsakoff syndrome (WIley and Gupta, 2020). Vitamin B12 (cobalamin) acts as a coenzyme, which is related to the syntheses of amino acids and cholesterol, with its deficiency resulting in neuropathy, demyelination, and dementia (Köbe et al., 2016; Clarke et al., 1998). Therefore, we hypothesized that dementia should occur more frequently in Japanese subjects who had lower serum vitamin B1 and B12 levels. If the association between the serum vitamin B level and dementia in Japanese could be determined, this would make it possible to decrease the risk of dementia by administering vitamin supplements and/or modification of the daily diet in Japan.

The aim of this current study was to investigate the association of vitamin B1 and B12 levels with dementia in Japanese subjects. Associations were evaluated for each sex, since hormones and functional biomarkers which vary with sex are considered to be risk factors of cognitive impairment (McDermott et al., 2017).

## Materials and Methods

### Subjects

The present study subjects included 222 patients who visited the Memory Clinic at Showa University Hospital, Tokyo, Japan from March 2016 to March 2019. Among these subjects, 221 patients agreed to participate in this study, with 1 patient not able to understand the Japanese required for completing the neuropsychological examination, while 28 lacked some of the data due to rejecting the tests. As a result, 192 participants (men: *n* = 65, women: *n* = 127) were analyzed in this study. All of the participants underwent routine blood and neuropsychological tests. The study and its consent procedure were approved by the Medical Ethics Committee of Showa University School of Medicine (approval no. 3088).

### Data Collection

Data were collected from the participants who visited the Memory Clinic. The Japanese version of the Mini-Mental State Examination (MMSE) and Hasegawa Dementia Scale-Revised (HDS-R) cognitive tests were administered by professionally trained neurologists. The scores ranged from 1 to 30, with the higher score indicating higher cognitive function.

MMSE is divided into two sections. The first section requires vocal response only and covers orientation, memory, and attention. The second part contains reading and writing sections such as writing a sentence and copying a figure, thereby covering both spatial cognitive and language functions. HDS-R is a test that is commonly used for screening dementia in Japan and consists of a vocal section only, which covers orientation, memory, attention and word fluency. Previous studies have defined dementia as a MMSE score ≤ 23 or HDS-R score ≤ 20, which were the scores that were used in our current study (Buckner, 2004; Araujo et al., 2015). Routine blood tests were performed for each participant in order to measure the vitamin levels. Vitamin B1 and B12 levels were measured by high performance liquid chromatography (HPLC) and clinical laboratory improvement amendments (CLIA) methods, respectively. The normal ranges of the vitamins were 21.3–81.9 mg/mL, 233–914 pg/mL, respectively (BML, Inc.).

### Statistical Analyses

Data are presented as median (25th percentile, 75th percentile) or n (%). Characteristics between the two groups (men vs. women and dementia group vs. non-dementia group) were compared using the Wilcoxon rank sum test for continuous variables and the Chi-squared test for categorical variables. Logistic regression analysis was used to calculate the odds ratio (OR) for dementia and the 95% confidence interval (CI). Age, vitamin B1 and vitamin B12, which were associated with dementia (Buckner, 2004; Araujo et al., 2015) were selected as potential confounders and included as explanatory variables in the model. A p value < 0.05 was considered as statistically significant. All statistical analyses were performed using JMP^®^ Pro 14 (SAS Institute Inc., Cary, NC, United States).

## Results

Table 1 presents the characteristics of study participants according to sex. No statistically significant differences were observed between men and women for the vitamin B1 and B12 levels. There were no statistically significant differences between men and women for dementia as defined by MMSE and HDS-R.

**TABLE 1:** Characteristics of study participants according to sex.

	Men *n* = 65	Women *n* = 127	p*
Age (years)	79 (74, 85)	81 (75, 85)	0.588
Vitamin B1 (ng/mL)	45.2 (38.6, 57.9)	42.8 (36.8, 52.5)	0.119
Vitamin B12 (pg/mL)	491.0 (381.5, 652.0)	479.0 (367.0, 658.0)	0.917
MMSE	23 (20, 27)	24 (19, 28)	0.443
HDS-R	21 (17, 25)	25 (18, 28)	0.444
Dementia defined by MMSE < 23, n (%)	35 (55.5)	58 (46.4)	0.236
Dementia defined by HDS-R < 20, n (%)	27 (42.9)	44 (35.2)	0.307

MMSE, Mini-Mental State Examination; HDS-R, Hasegawa Dementia Rating Scale-Revised. Values are expressed as the median (25th percentile, 75th percentile) except where n (%) is indicated. *Wilcoxon rank-sum test or Chi-squared test.

We initially assessed differences in the serum vitamin B levels between the dementia and non-dementia groups diagnosed based on MMSE scores. As shown in Table 2, the dementia patients were significantly older than the non-dementia patients. In addition, the dementia group had significantly lower serum levels of vitamin B1 and B12 as compared to the non-dementia patients. When we diagnosed patients according to the HDS-R, the dementia patients were also older and had lower serum vitamin B1 levels, while the difference for the serum vitamin B12 level was marginally significant.

**TABLE 2:** Characteristics of dementia and non-dementia group.

	Dementia defined by MMSE ≤ 23			Dementia defined by HDS-R ≤ 20		
		
	Dementia *n* = 95	Non-Dementia *n* = 97	p*	Dementia *n* = 71	Non-Dementia *n* = 121	p*
Age (years)	82 (76, 86)	78 (73, 84)	0.003	82 (77, 86)	79 (74, 84)	0.017
Vitamin B1 (ng/mL)	42.6 (34.8, 51.2)	45.2 (40.6, 55.7)	0.005	40.6 (34.1, 47.5)	45.2 (40.4, 56.8)	< 0.001
Vitamin B12 (pg/mL)	448.0 (347.0, 599.0)	516.0 (395.0, 690.0)	0.019	462.0 (333.0, 590.0)	507.0 (386.0, 681.0)	0.056
MMSE	19 (15, 22)	27 (26, 29)	< 0.001	18 (15, 21)	26 (24, 29)	< 0.001
HDS-R	17 (13, 21)	27 (25, 29)	< 0.001	16 (12, 18)	26 (24, 29)	< 0.001

MMSE, Mini-Mental State Examination; HDS-R, Hasegawa Dementia Rating Scale-Revised. Values are expressed as the median (25th percentile, 75th percentile) except where n (%) is indicated. *Wilcoxon rank-sum test or Chi-squared test.

Subsequently, we then examined whether these differences were characterized according to sex. Table 3 presents the comparisons of age, vitamin B1 and vitamin B12 between the dementia group (MMSE ≤ 23) and non-dementia group (MMSE > 23) according to sex. There were no statistically significant differences for the age and vitamins among men. In contrast, there were statistically significant differences for both the age and vitamins observed among women, with the dementia group older than the non-dementia group (*p* = 0.003), while the vitamin B1 and vitamin B12 levels were significantly lower in the dementia group (*p* = 0.001 and 0.012, respectively) versus the non-dementia group. Similar results were obtained even when the dementia was determined by HDS-R ≤ 20 (Table 4).

**TABLE 3:** Comparisons of characteristics between the dementia group (MMSE ≤ 23) and the non-dementia group (MMSE > 23) according to sex.

		Dementia	Non-Dementia	p*

Men		*n* = 36	*n* = 29	
	Age (years)	79.0 (74.3, 85.8)	78.0 (73.0, 84.0)	0.311
	Vitamin B1 (ng/mL)	44.8 (35.3, 62.3)	45.3 (41.3, 55.6)	0.644
	Vitamin B12 (pg/mL)	484.0 (363.0, 639.0)	491.0 (399.5, 673.0)	0.787

**Women**		**n = 59**	**n = 68**	

	Age (years)	82.0 (78.0, 86.0)	78.0 (73.0, 83.0)	0.003
	Vitamin B1 (ng/mL)	40.6 (34.1, 47.5)	45.0 (40.5, 59.4)	0.001
	Vitamin B12 (pg/mL)	423.0 (318.0, 579.0)	551.0 (395.0, 739.8)	0.012

MMSE: Mini-Mental State Examination. *Wilcoxon rank-sum test.

**TABLE 4:** Comparisons of characteristics between the dementia group (HDS-R ≤ 20) and the non-dementia group (HDS-R > 20) according to sex.

		Dementia	Non-dementia	p*

Men		*n* = 27	*n* = 38	
	Age (years)	80.0 (75.0, 86.0)	77.0 (74.0, 84.3)	0.195
	Vitamin B1 (ng/mL)	43.0 (34.8, 58.4)	47.3 (42.3, 57.1)	0.074
	Vitamin B12 (pg/mL)	470.0 (354.0, 664.0)	496.0 (381.8, 647.0)	0.952

**Women**		***n = 44***	***n = 83***	

	Age (years)	82.0 (77.3, 86.8)	80.0 (74.0, 84.0)	0.041
	Vitamin B1 (ng/mL)	39.1 (34.1, 46.3)	44.9 (40.2, 56.7)	0.001
	Vitamin B12 (pg/mL)	421.5 (310.5, 585.8)	511.0 (390.0, 740.0)	0.033

HDS-R, Hasegawa Dementia Rating Scale-Revised. *Wilcoxon rank-sum test.

Table 5 showed ORs for dementia (MMSE ≤ 23) according to the tertiles of age, vitamin B1 and vitamin B12. When compared to the lowest age and highest vitamin level group (third tertile; T3), ORs of the first tertile (T1) and second tertile (T2) for vitamin B1 and vitamin B12 were not statistically significant among men. In contrast, there were significantly increased ORs of the T1 for age, vitamin B1 and B12 found among women. Even after adjustment for potential confounders, there was still a significantly increased OR of the T1 for vitamin B1. These findings were similar even when dementia was defined by HDS-R ≤ 20 (Table 6).

**TABLE 5:** Associations of vitamin B1 and vitamin B12 with dementia defined by MMSE ≤ 23 in each sex.

		Total *n*	Dementia n (%)	Crude		Adjusted*	
		
				OR (95% Cl)	*p*	OR (95% Cl)	*p*
**Men**							
**Age (years)**							
	T1 (83–98)	24	14 (58.3)	1.56 (0.46–5.23)	0.475	0.24 (0.19–3.01)	0.268
	T2 (75–83)	22	13 (59.1)	1.60 (0.47–5.54)	0.454	0.72 (0.15–3.50)	0.684
	T3 (51–75)	19	9 (47.4)	1.00		1.00	
**Vitamin B1 (ng/mL)**							
	T1 (24.4–42.2)	21	14 (66.7)	1.38 (0.40–4.80)	0.608	1.37 (0.39–4.87)	0.623
	T2 (42.2–53.0)	22	9 (40.9)	0.50 (0.14–1.59)	0.230	0.53 (0.15–1.85)	0.318
	T3 (53.0–412.8)	22	13 (59.1)	1.00		1.00	
**Vitamin B12 (pg/mL)**							
	T1 (105–412.8)	21	12 (57.1)	0.92 (0.27–3.10)	0.897	0.92 (0.26–3.23)	0.894
	T2 (412.8–588.0)	22	11 (50.0)	0.69 (0.21–2.28)	0.545	0.64 (0.19–2.16)	0.468
	T3 (588.0–1455.0)	22	13 (59.1)	1.00		1.00	
**Women**							
**Age (years)**							
	T1 (83–93)	46	27 (58.7)	2.95 (1.22–7.15)	0.017	1.32 (0.21–8.33)	0.767
	T2 (77–83)	41	19 (46.3)	1.79 (0.73–4.42)	0.205	1.24 (0.33–4.69)	0.747
	T3 (50–77)	40	13 (32.5)	1.00		1.00	
**Vitamin B1 (ng/mL)**							
	T1 (4.6–39.7)	42	28 (66.7)	3.73 (1.52–9.16)	0.004	2.59 (1.00–6.70)	0.049
	T2 (39.7–47.5)	42	16 (38.1)	1.15 (0.47–2.78)	0.759	0.81 (0.31–2.08)	0.655
	T3 (47.5–282.0)	43	15 (34.9)	1.00		1.00	
**Vitamin B12 (pg/mL)**							
	T1 (105.0–401.2)	42	24 (57.1)	2.97 (1.22–7.28)	0.017	2.28 (0.89–5.85)	0.088
	T2 (401.2–591.4)	43	22 (51.2)	2.34 (0.96–5.67)	0.061	2.31 (0.91–5.89)	0.079
	T3 (591.4–3733.0)	42	13 (31.0)	1.00		1.00	

MMSE, Mini-Mental State Examination; T1, tertile 1; T2, tertile 2; T3, tertile 3. *Age, vitamin B1, and vitamin B12 were included as explanatory variables in a logistic regression model.

**TABLE 6:** Associations of vitamin B1 and vitamin B12 with dementia defined by HDS-R ≤ 20 in each sex.

		Total *n*	Dementia n (%)	Crude		Adjusted*	

				OR (95% Cl)	*p*	OR (95% Cl)	*P*
**Men**							
**Age (yaers)**							
	T1 (83–98)	24	11 (45.8)	1.83 (0.52–6.44)	0.345	0.37 (0.03–5.32)	0.461
	T2 (75–83)	22	10 (45.5)	1.81 (0.50–6.50)	0.366	0.87 (0.17–4.41)	0.871
	T3 (51–75)	19	6 (31.6)	1.00		1.00	
**Vitamin B1 (ng/mL)**							
	T1 (24.4–42.2)	21	13 (61.9)	2.84 (0.83–9.80)	0.098	3.08 (0.86–11.02)	0.084
	T2 (42.2–53.0)	22	6 (27.3)	0.66 (0.18–2.36)	0.519	0.80 (0.21–3.02)	0.739
	T3 (53.0–412.8)	22	8 (36.4)	1.00		1.00	
**Vitamin B12 (pg/mL)**							
	T1 (105–412.8)	21	8 (38.1)	0.89 (0.26–3.02)	0.850	0.75 (0.21–2.70)	0.658
	T2 (412.8–588.0)	22	10 (45.5)	1.20 (0.36–3.97)	0.761	1.10 (0.32–3.76)	0.882
	T3 (588.0–1455.0)	22	9 (40.1)	1.00		1.00	
**Women**							
**Age (years)**							
	T1 (83–93)	46	19 (41.3)	1.22 (0.51–5.33)	0.114	1.32 (0.19–9.19)	0.776
	T2 (77–83)	41	15 (36.6)	1.73 (0.66–4.50)	0.261	1.40 (0.34–5.73)	0.641
	T3 (50–77)	40	10 (25.0)	1.00		1.00	
**Vitamin B1 (ng/mL)**							
	T1 (4.6–39.7)	42	22 (52.4)	3.63 (1.43–9.21)	0.007	2.75 (1.03–7.38)	0.044
	T2 (39.7–47.5)	42	12 (28.6)	1.32 (0.50–3.50)	0.576	1.01 (0.36–2.84)	0.982
	T3 (47.5–282.0)	43	10 (23.3)	1.00		1.00	
**Vitamin B12 (pg/mL)**							
	T1 (105.0–401.2)	42	19 (45.2)	2.64 (1.04–6.73)	0.042	1.96 (0.74–5.18)	0.174
	T2 (401.2–591.4)	43	15 (34.9)	1.71 (0.66–4.42)	0.265	1.60 (0.59–4.29)	0.353
	T3 (591.4–3733.0)	42	10 (23.8)	1.00		1.00	

HDS-R, Hasegawa Dementia Rating Scale-Revised; T1, tertile 1; T2, tertile 2; T3, tertile 3. *Age, vitamin B1 and vitamin B12 were included as explanatory variables in a logistic regression model.

## Discussion

Our current study demonstrated that serum vitamin B1 levels were negatively associated with dementia development in women. This result suggests that the influence of vitamin B1 on dementia differs between men and women. To our knowledge, this is the first study to examine whether the relationship between serum vitamin B level and dementia risk differs according to sex. However, the present study findings need to be carefully evaluated.

Vitamin B1 (thiamin) deficiency leads to both central and peripheral nerve dysfunctions, including cognitive impairment such as Wernicke-Korsakoff syndrome (WKS) (Gibson et al., 2016; Yu et al., 2018). However, even within the normal range of vitamin B1, our study showed that a lower vitamin level increased the risk for dementia. Furthermore, a previous study suggested that the vitamin B1 level and cognitive impairment were involved with ApoEε2, although it is believed that ApoEε4 is more responsible for the cognitive impairment (Lu et al., 2015). An additional study examined the relationship between WKS and the ApoE allele. This study found that WKS patients with a low full-score IQ (FIQ), which was tested by the Wechsler Adult Intelligence Scale Revised version (WAIS-R), exhibited a higher frequency of ApoEε4 (Muramatsu et al., 1997). ApoE is a known factor for apolipoprotein nervous tissue, with an involvement in the mobilization and redistribution of cholesterol in the repair, growth, and maintenance of myelin and neuronal membranes. Recently, ApoEε4 has received a lot of attention, as it is one of the genetic risk factors for late-onset Alzheimer’s disease, with women having a higher risk for developing cognitive decline, although the involved mechanism has yet to be determined (Altmann et al., 2014; Vardarajan et al., 2014; Dubal and Rogine, 2017). Vitamin B12 deficiency has been shown to be associated with various neurodysfunctions, such as polyneuropathy and damage to the white matter in the spinal cord and brain (Shipton and Thachil, 2015; Miller et al., 2005; Scherer, 2003). In addition, it has also been reported that this association may only exist in ApoEε4 carriers (Lee et al., 2016). While the exact mechanism remains unclear, the leading hypothesis is that demyelination occurs due to incompetence of methylation of the myelin basic protein (MBP) (O’Leary et al., 2012). MBP consists of 30% of myelin protein, which covers the axon of the nerve cell and speeds up the neural transmission (Boggs, 2006). Therefore, a low vitamin B12 status can impair the methionine synthesis and lead to S-adenosylmethionine deficiency, which results in demethylation of myelin phospholipid in the central nervous system. In addition, the basis of the homocysteine theory is that a vitamin B12 deficiency can lead higher homocysteine levels in the serum, which is an important risk of atherosclerosis (Ostrakhovitch and Tabibzadeh, 2019; McCully and Wilson, 1975). In fact, some studies have reported finding that an elevated homocysteine level increases the risk for Alzheimer’s disease (Seshadri et al., 2002; Morris, 2012; Morris et al., 2006). Thus, this potential mechanism may be involved in the risk for these types of cognitive impairments. However, we could find no reasonable explanation as to why the ORs for vitamin B12 lost their significant difference after being adjusted for vitamin B1.

Many previous studies have revealed the association between vitamins and dementia (Travica et al., 2017; O’Leary et al., 2012; Balbaloglu and Tanik, 2019; Gibson et al., 2016; Hughes et al., 2017). However, there have been few studies that have examined differences in the sex with regard to the relationship between the vitamin level and dementia risk. Alzheimer’s disease, which accounts for over half of all dementia, occurs at a greater frequency in women versus men. A study shows the higher vitamin C level reduce the risk of cognitive decline in women with APOE4 and men without APOE4 (Noguchi-Shinohara et al., 2018). Factors related to the female endocrine system are assumed to be associated with the differences observed according to the sex. Additionally, although it has also been reported that being an ApoE4 carrier is a risk factor for Alzheimer’s disease, especially in women, this mechanism has yet to be definitely determined (Vardarajan et al., 2014; Dubal and Rogine, 2017; Stanhewicz et al., 2018). Another possible explanation could be associated with a vascular factor. Vascular risk factors such as hypertension, hyperlipidemia, diabetes mellitus, being overweight, and smoking are known to enhance the risk of dementia (Blom et al., 2013). Indeed, patients with Alzheimer’s disease commonly show cerebrovascular dysfunction. Large-scale autopsy studies indicated that there was a greater burden of macro- and microinfarcts, atherosclerosis, arteriosclerosis, and cerebral amyloid angiopathy (CAA) in Alzheimer’s disease as compared to other neurodegenerative diseases (Toledo et al., 2013). Furthermore, it has also been reported that there is an increased Alzheimer’s disease risk in cases with infarcts and more severe atherosclerosis or arteriosclerosis (Arvanitakis et al., 2016). A recent neuroimaging study demonstrated that the serum level of HDL cholesterol, which is one of the vascular risk factors, was associated with not only preserved cognitive function but also gyrification of the insular and frontal opercular cortex (Kinno et al., 2019). These findings demonstrated that there was an association between better vascular health and decreased Alzheimer’s disease risk. The importance vitamin B12 in the reduction of the risk of vascular disease is well-known (Quinlivan et al., 2002). Our current results suggest that the vasoprotective effects of vitamin B12 on cognitive function are more prominent for women versus men.

There were several limitations for our current study. First, we did not take into consideration the participants’ past health history such as hypertension, diabetes, and dyslipidemia. Since these diseases have been reported to be associated with dementia (Turana et al., 2019; Benn et al., 2017; Koch and Jensen, 2016; Zheng et al., 2018; Fan et al., 2017), this could have affected our current study findings. For the same reason, the history of life which associate with dementia, such as medication, nutrition supplement and educational periods, are not considered in this study (Tucker and Stern, 2011). Second, because this study was a cross-sectional study, it cannot be definitively determined whether or not the vitamin deficiency causes dementia. Therefore, future prospective studies will need to be undertaken in order to determine if the vitamin deficiency occurs prior to the cognitive decline. Third, our study was conducted at an outpatient clinic of one university hospital in Tokyo, Japan. All the participants were conscious of memory loss, were able to make the decision to visit the hospital, and had great enough economic resources in order to be seen by the neurologists at a university hospital instead of only their primary care doctor. Thus, our participants might not reflect the general characteristics of the overall population in Japan.

## Conclusion

The present study showed that serum vitamin B1 levels were associated with dementia only in women. These results suggest that vitamin B1 may have a role in helping to prevent the development of dementia in women. Future longitudinal studies will need to be undertaken in order to definitively determine whether a decreasing vitamin level occurs prior to cognitive impairment, and whether maintaining a higher vitamin level can prevent a worsening of the cognitive function and the subsequent development of dementia.

## Data Availability Statement

The raw data supporting the conclusions of this article will be made available by the authors, without undue reservation.

## Ethics Statement

The studies involving human participants were reviewed and approved by Medical Ethics Committee of Showa University School of Medicine. The patients/participants provided their written informed consent to participate in this study.

## Author Contributions

AM and HO planned the present study. RK, AK, and KO contributed to improving the study in a meaningful way. AM drafted the manuscript. AM, RK, SK, YM, AF, AS, TK, HK, SY, and SH performed data collection. RK, HO, and KO supported the draft of this manuscript and data collection. AM, HO, and AK contributed to the statistical analysis. RK, HO, AS, TK, SH, and KO made the revision of the manuscript. All authors read and approved the final manuscript.

## Conflict of Interest

The authors declare that the research was conducted in the absence of any commercial or financial relationships that could be construed as a potential conflict of interest.
